# A Crime by Any Other Name: Gender Differences in Moral Reasoning When Judging the Tax Evasion of Cryptocurrency Traders

**DOI:** 10.3390/bs14030198

**Published:** 2024-03-01

**Authors:** Jori Grym, Jaakko Aspara, Monomita Nandy, Suman Lodh

**Affiliations:** 1 Department of Marketing, Hanken School of Economics, 00100 Helsinki, Finland; jaakko.aspara@hanken.fi; 2 Department of Business and Management, LUISS Guido Carli, 00197 Roma, Italy; 3 College of Business, Arts and Social Sciences, Brunel University London, Middlesex UB8 3PH, UK; monomita.nandy@brunel.ac.uk; 4 Kingston Business School, Kingston University London, Kingston upon Thames KT1 2EE, UK; s.lodh@kingston.ac.uk

**Keywords:** tax morale, gender differences, moral foundations, tax evasion, cryptocurrency, judgment

## Abstract

Tax evasion is a major issue for authorities worldwide. Understanding the factors that influence individuals’ intrinsic motivation to pay taxes, known as their tax morale, is important for improving tax compliance. This study investigated gender differences in judging tax evasion in the context of cryptocurrency trading. Specifically, a survey study explored whether different moral foundations, financial literacies, and political orientations among females vs. males might explain potential gender differences in judging tax evasion. In an online survey, 243 U.S. adults read a vignette about a friend evading taxes in a cryptocurrency trading context. In a correlational analysis, we found that females judged tax evasion harsher, as being more morally wrong than males. Of the psychographic factors, only individualizing moral foundation values (i.e., fairness and harm avoidance) explained the harsher moral judgment by females. That is, individualizing moral foundation values were at a higher level among females, which further predicted females’ harsher judgment of tax evasion. While females also had, on average, lower financial literacy and knowledge of cryptocurrencies than males, these did not predict their harsher judgment of tax evasion. The findings contribute to research on gender differences in moral judgments and highlight that a given transgression, or a specific crime, may violate different moral values in men and women. The results demonstrate to policy makers that it is important to take into account gender differences, in campaigns promoting tax morale and compliance.

## 1. Introduction

Tax morale—an individual’s intrinsic motivation to pay their taxes and/or avoid tax evasion [1,2]—is a topic of growing interest in behavioral finance [3] and accounting [4] research as well as the literature on taxation and law [5]. Besides being an intriguing phenomenon in its own right, understanding the factors that influence citizens’ tax morale is crucial for improving tax policies, and public policies and societal welfare in general [6]. Tax compliance vs. evasion is also significantly correlated with economic growth [7], and tax evasion is a type of economic crime (refer to [8] for a definition and the types of economic crime). Moreover, it is increasingly important and topical to investigate factors affecting tax morale (vs. evasion), as emerging new technologies such as digital cryptocurrencies provide citizens with new kinds of labor and capital income opportunities, and paying due taxes for these incomes often rests on the conscience of the individual income earner [9].

However, extant literature on cryptocurrencies and the benefits and drawbacks of cryptocurrencies in facilitating tax evasion is inconclusive [10]. Among factors mentioned by scholars as influencing tax evasion in the context of cryptocurrency trading are, for instance, the lack of well-defined tax compliance structure [11] and the easiness of cross-border transactions [12]. Still, extant research on tax evasion in the cryptocurrency context has not yet explored the impact of such factors that are commonly known to influence individuals’ tax morale in general, such as gender.

Indeed, recent research on tax morale in general has paid increasing attention to the role of gender differences. Overall, females seem to exhibit a higher tax morale compared to males (e.g., [13,14,15,16]). Furthermore, Hasseldine and Hite [17], for instance, investigated framing effects on tax compliance and found a significant interaction effect between message framing and gender. This demonstrates that gender also plays a role in how messages about tax compliance are processed and acted upon. 

Nevertheless, while indicating various gender differences in tax morale, previous studies have fallen short of unpacking the underlying psychographic factors that give rise to those gender differences—whether in general or in the particular context of cryptocurrencies. To address this research gap, the present research asks the following research questions: (1) Are there gender differences in tax morale when it comes to income from a new digital asset, cryptocurrency? (2) Can the gender differences in tax morale be explained by the differences in certain psychographic factors across females vs. males?

As to the psychographic factors, the present research focuses on attitudinal “moral foundations”, political orientation (liberalism vs. conservatism), and financial knowledge or literacy. These psychographic factors may be further affected by inequalities in governance systems, which are responsible for the tax crimes within national boundaries and beyond [18]. Of these, financial literacy has been shown to affect many financial behaviors, for instance estimation of the financial values of annuities, participation in investment markets, and risk-taking (e.g., [19,20,21,22]). Also, males have been found to be more financially literate than females, on average (e.g., [23,24,25,26]). Political orientation may also affect financial decision making (e.g., [27,28]), and females are known to be generally more liberal than males [29]. In turn, attitudinal moral foundations are chronic attitudes that individuals hold about fundamental moral values and principles, which in turn guide the individual’s moral judgments and behaviors [30]. As such, it is conceivable that individuals’ moral foundation values may also affect their tax morale, and individuals of different genders have, on average, different emphases in their moral foundations [31].

To investigate whether potential gender differences in tax morale with respect to cryptocurrency investing can be explained by gender differences in moral foundation values, political orientation, and financial literacy, we conducted a survey among U.S. consumers (n = 243), recruited from Amazon MTurk. In the survey, we presented the respondents with a scenario in which their friends were evading taxes on cryptocurrency trading income. The dependent variable was the perceived moral wrongness of said tax evasion. As to the results, we find that female respondents perceive tax evasion as more wrong than males, on average. Furthermore, of the psychographic factors, we find that only “individualizing” moral foundation values (e.g., [30]) significantly explain the effect of gender on moral judgments of tax evasion. That is, females assign, on average, higher moral value to individual fairness and the protection of individual rights, and this in turn correlates with their harsher judgment of the wrongness of tax evasion. While we also find gender differences in general financial literacy and the knowledge of cryptocurrencies, these variables do not further predict the moral judgment of tax evasion and cannot therefore explain the gender difference in tax morale.

The present research contributes to several areas of research. First, in terms of research on gender differences in tax morale, we extend recent research [13] that has speculated that females and males may employ distinct moral values when judging tax issues. Confirming this conjecture, the present research shows that females tend to exhibit a higher degree of “individualizing” moral foundation values especially, and that these values further predict females’ more negative moral judgment of tax evasion. As another contribution to the research on the gender differences in tax morale, the present study is, to our knowledge, the first one to focus on tax morale related to the new income opportunities brought about by contemporary technology-led digital assets (such as cryptocurrency), instead of only addressing general tax morale.

Second, our results contribute to general behavioral research on moral foundations. Our results are consistent with the observations of previous research (e.g., [31]) in finding that females generally draw more on individualizing moral foundations than males. However, our new finding is that females’ higher individualizing moral foundation values in general also further predict their moral judgments in a particular domain of the economy and financial behavior: tax morale.

Third, the present research contributes to the growing body of behavioral research on cryptocurrency investing [32,33,34]. Regarding this literature, the present study is, to our knowledge, the first to examine individuals’ moral judgments vis à vis cryptocurrencies, rather than basic cognitive variables (e.g., familiarity with cryptocurrencies) and behavioral outcomes (e.g., participation in cryptocurrency markets).

Fourth, to explain the gender differences in tax morale, we extend all the aforementioned streams of research by testing three alternative psychographic variables in one model. Fifth, and finally, the findings of this study draw guidelines for regulators developing governance mechanisms that are applicable in the context of digital assets, like cryptocurrency.

In the following sections, we summarize the relevant literature, and describe the method used in this research. In the last two sections, we present the results and discuss our findings to draw the conclusions of the study, indicating the direction of future research.

## 2. Literature Review

### 2.1. Tax Morale and Gender Differences

Broadly, tax morale refers to the intrinsic motivation and willingness of taxpayers to comply with tax laws and pay their taxes [2]. It is an important factor influencing voluntary compliance with the tax system. Tax evasion involves the illegal nonpayment or underreporting of taxes owed [1].

Overall, research indicates that women tend to exhibit higher tax morale and compliance compared to men (e.g., [13,14,15,16]). For example, a meta-analysis of survey studies from 111 countries found a consistent result: females have more negative attitudes towards tax evasion and view it as less acceptable compared to males, and display greater intrinsic motivation to pay taxes across cultures [15]. 

However, few studies address the potential reasons for such a gender difference in tax morale, in terms of psychographic factors or traits. In other words, there is little research on whether certain cognitive or attitudinal traits or values—the mean levels of which may differ across genders—may explain the difference in the average tax morale of females vs. males. As an exception, a recent study by Rahmawati and Dwijayanto [13] proposed that the logic behind the tax morales of men and women may systematically differ. In their study in Indonesia, they found that women’s tax ethics centered more on moral values, while men focused more on logical reasoning (e.g., likelihood of getting caught for tax evasion). This suggests that gender differences in tax morale may indeed arise from women and men exhibiting different moral attitudes and other psychographic factors when it comes to taxation matters and tax evasion. In the following section, we speculate on three alternative psychographic variables which might explain gender differences in tax morale.

### 2.2. The Alternative Psychographic Variables Explaining the Gender Differences in Tax Morale

#### 2.2.1. Moral Foundations

The first psychographic variables of interest at present are moral foundations. Moral foundations are individual-level, chronic moral attitudes or values on which individuals make moral judgments [35,36,37]. Two main classes of moral foundation attitudes have been identified in previous research [30]. The first class is “binding” moral foundations, which reflect the extent to which an individual morally values loyalty to groups and communities, is committed to moral purity, and respects authorities. The second class is “individualizing” moral foundations, which reflect the extent to which an individual assigns moral value to individual fairness, to the protection of individual rights, and to the avoidance of harm.

Relating to how individuals make moral judgments in general, it is likely that binding and individualizing moral foundations may also be associated with individuals’ tax morale and moral judgments of tax evasion. What makes moral foundation values especially interesting to the present research questions is that fact that prior research has observed gender differences in moral foundations as well [38]. That is, when making moral judgments, women appear to draw more on individualizing moral foundations, while men weigh binding foundations more heavily [31]. Related earlier research also indicates that women tend to emphasize care, empathy, and universal welfare in their moral judgments, while men focus on justice, social order, and reciprocity norms more frequently [39].

Given the fact that individuals’ moral foundation values likely affect their tax morale as well, and the fact that there are gender differences in their moral foundation values, it is conceivable that part of the gender differences in tax morale could be explained by gender differences in moral foundation values.

#### 2.2.2. Financial Literacy

As a common control variable in behavioral finance research [21,25,27], financial literacy refers to an individuals’ knowledge and experience of financial instruments and markets. Studies have found gender differences both in individuals’ general financial literacy and in their knowledge of particular financial instruments. Both general financial literacy and literacy about a particular financial instrument could affect an individual’s tax morale. For instance, regarding the latter, if the individual is not at all familiar with using an instrument (e.g., cryptocurrency), they may not “know”, either, that one should pay taxes for gaining income from that instrument.

With regard to general financial literacy, males have often been observed to have more knowledge and experience of financial markets than females (e.g., [23,24,25,26]). Furthermore, this gender difference tends to become emphasized with more special, high-tech (or ‘fintech’), and innovative financial instruments. As such, males have also been found to have higher knowledge and literacy about contemporary financial instruments such as cryptocurrency [40].

Thus, as financial literacy may affect tax morale, and as financial literacy often differs across genders, gender differences in tax morale may also be partly explained by differences in financial literacy. In the present study, we will measure gender differences in both financial literacy in general and financial literacy about cryptocurrencies in particular.

#### 2.2.3. Political Orientation

The third psychographic factor which we will presently focus on is political orientation. Defined as an individual’s orientation towards liberal vs. conservative values and political parties, political orientation has also been found to affect individuals’ financial judgments and decisions (e.g., [27,41]). Moreover, political orientation has been shown to affect, and interact with, the aforementioned moral foundation values and moral judgments [42]. At the same time, women have often been observed to exhibit, on average, more liberal political values than males [29,43,44]. Thus, since political orientation may affect financial and moral judgments—and potentially also tax morale—and since there are gender differences in the political orientation of individuals, it may be that the gender differences in tax morale are also partly explainable by the gender differences in political orientation.

## 3. Method

### 3.1. Sample

This study employed a correlational survey design, utilizing a sample of respondents recruited from Amazon Mechanical Turk (MTurk). MTurk is an online crowdsourcing platform that allows researchers to recruit respondents for survey studies from an online respondent pool. The respondent pool of MTurk has been shown to adequately represent the general population in the United States in terms of psychological traits [45]. The use of MTurk as a source of survey respondents for correlational research has become increasingly common in various fields, including psychology, marketing, and behavioral economics [46,47]. The data were gathered between December 2019 and January 2020.

Consent from respondents was established by a form that clearly stated the purpose of the study and how the data would be processed and used, with the options of “I consent” vs. “I do not consent”.

A total of 243 qualified and complete responses were received and subjected to the analyses below, after 8 responses were disqualified due to failing an instructive attention check and 3 responses for not completing the survey in its entirety.

In total, 55% of the respondents were male and 45% female. The age of the respondents ranged from 21 to 77 years, with a mean of 39.6 years. Especially when it comes to the main predictor variable in this study, female vs. male gender, a question arises of whether the study’s sample adequately represented the U.S. population in terms of gender. The descriptive statistics above indicate that the representativeness of the sample in terms of the two genders was not very good, as 55% of the sample was male, while above 50% of the U.S. population is female [48]. However, this does not necessarily bias the present results considerably for two reasons. First, the present results essentially focus on the differences in the mean judgments of tax evasion in the sub-samples of female vs. male respondents, rather than the overall means of judgments in the entire sample including males and females. Thus, even if there is a higher proportion of males in the sample than in the U.S. population, meaning that the overall means are likely to be biased towards males’ judgments, this does not signify that the means within the sub-samples of males vs. females are considerably biased. Second, we have no reason to believe that willingness to participate in the survey, among males vs. females, would somehow depend on the studied variables (e.g., judgments of tax evasion). This is because the exact variables under study were not yet revealed to the respondents at the time when they decided to start responding to the survey. Thus, differences in the means of the studied variables across the two genders are not likely to be due to the fact that morally more vs. less judgmental individuals would have had differential willingness to participate in the survey across the male vs. female sub-samples.

### 3.2. Measurements

In the survey questionnaire, the respondents were presented with a short vignette, describing a friend that engages in tax evasion after making profitable trades in cryptocurrencies:

“You meet a friend for lunch. They tell you about a new investment website online that operates in another country. During the last year they got a hang of trading and started making steady profits on their investments. Eventually they concentrated all their investments in cryptocurrencies such as bitcoin since those seemed to yield the greatest profits.

By the end of the year, they made 100% profit on their initial investment. The website does not provide automatic tax reporting to the government, and your friend decides not to inform the tax authorities on the profits.”

After reading the above vignette, the respondents were asked a question pertaining to the main dependent variable, their moral judgment of tax evasion. Specifically, we utilized a question with three items, asking “how wrong”, “how immoral”, and “how morally wrong” the actions of the friend depicted in a vignette were, in the respondent’s opinion. Responses were recorded on a 7-point semantic differential scale (1 = Did not act wrong, 7 = Acted very wrong). Similar questions have been used in earlier studies related to moral judgments (e.g., [30,49,50]). Cronbach’s alpha for this three-item question was high, at α = 0.902.

After the dependent variable question, the respondents were presented with questions pertaining to the main psychographic variables: their moral foundation values, financial literacy, and political orientation.

For moral foundation values, the question items were adopted from the Moral Foundations Questionnaire (MFQ) [30]. For “binding” moral foundations, the following nine questions were asked, on a 7-point scale (1 = strongly disagree, 7 = strongly agree): “I am proud of my country’s history”, “People should be loyal to their family members, even when they have done something wrong”, “It is more important to be a team player than to express oneself”, “Respect for authority is something all children need to learn”, “Men and women each have different roles to play in society”, “If I were a soldier and disagreed with my commanding officer’s orders, I would obey anyway because that is my duty”, “People should not do things that are disgusting, even if no one is harmed”, “I would call some acts wrong on the grounds that they are unnatural”, and “Chastity is an important and valuable virtue”.

For “individualizing” moral foundations, the respondents were presented with the following six questions: “Compassion for those who are suffering is the most crucial virtue”, “One of the worst things a person could do is hurt a defenseless animal”, “It can never be right to kill a human being”, “When the government makes laws, the number one principle should be ensuring that everyone is treated fairly”, “Justice is the most important requirement for a society”, and “I think it’s morally wrong that rich children inherit a lot of money while poor children inherit nothing.” [30]. The reliability of both constructs was satisfactory in terms of their Cronbach’s alpha (α_Binding moral foundations_ = 0.89; α_Individualizing moral foundations_ = 0.62).

Of the other psychographic variables, political orientation was measured using three items, as follows: “Where on the following scale of political orientation would you place yourself (overall, in general)?” “In terms of social and cultural issues in particular, how liberal or conservative are you?” and “In terms of economic issues in particular, how liberal or conservative are you?” The responses to all the items were recorded on a 7-point scale from 1 = “extremely liberal” to 7 = “extremely conservative” [51]. The reliability of the construct was again high (α_Political orientation_ = 0.94).

Lastly, financial literacy about cryptocurrencies was measured by asking the respondents “how well do you understand how cryptocurrency works?” Their responses were recorded on a 9-point scale (1 = “I do not understand how cryptocurrency investing works”, 9 = “I fully understand how cryptocurrency investing works”). In turn, general financial literacy was established through a similar question item, which replaced the “cryptocurrency investing” above with “stock investing”. These scales were converted to a 7-point scale for analysis, like the other variables.

In addition to the above psychographic variables, we included questions about a set of demographic variables. These included the main predictor variable, gender, as well as the following control variables: age, education level, and income level.

When it comes to multi-item measures (i.e., individualizing moral foundations, binding moral foundations, political orientation, tax morale), we used—as variable values in descriptive analyses as well as linear regressions (reported in Appendix B)—the average value of a respondent’s responses to the question items. For instance, for tax morale, which was measured using three question items, the mean value of a given respondent’s responses to these three items was used as the variable value. However, in the partial least squares (PLS) analysis, the latent factor scores estimated by the PLS model were used as variables.

## 4. Results

Table 1 below shows the descriptive statistics of the studied variables for female vs. male respondents, respectively. As is visible in the table, females judged tax evasion to be significantly more wrong than males (M_females_ = 5.22; M_males_ = 4.50; t = −3.70, *p* < 0.001).

Of the moral foundation values, females put more weight, on average, on individualizing moral foundations than males (M_females_ = 4.65; M_males_ = 4.45; t = –2.05, *p* < 0.04). In terms of binding moral foundations, there was no significant difference between males and females. In contrast, for both general financial literacy (M_females_ = 4.79; M_males_ = 5.77; t = 3.64, *p* < 0.001) and literacy about cryptocurrencies (M_females_ = 4.05; M_males_ = 5.63; t = 5.19, *p* < 0.001), males had higher average literacy than females. Again, there was no significant difference between males and females in political orientation (conservatism vs. liberalism).

The descriptive statistics and pairwise *t*-tests in Table 1 imply that the most potent psychographic variables that explain the gender difference in tax morale are individualizing moral foundation values as well as financial literacy. Table 2 shows the bivariate correlations between the variables.

**Figure 1:**
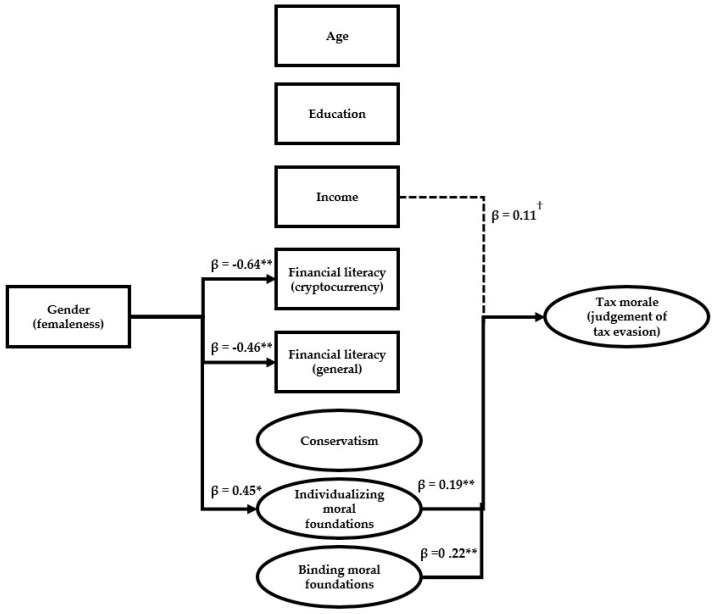
Results of the PLS path model. Notes: only the statistically significant effect paths are included in the figure. ^†^ *p* < 0.10, * *p* < 0.05, ** *p* < 0.01.

In Figure 1, we estimate a partial least squares (PLS) path model to further analyze the relationships between the variables. Given the large number of path effects estimated, Figure 1 only includes the path coefficients (and ‘arrows’) that were statistically significant in the analysis.

The bootstrapping procedure in SmartPLS involves repeatedly sampling from the original data, using replacement to create a large number of bootstrap samples (e.g., 5000 samples). For each bootstrap sample, SmartPLS estimates the path model parameters using the PLS algorithm [52]. The standard errors for each model parameter are then calculated as the standard deviation of the corresponding bootstrap estimates across all bootstrap samples [53].

To calculate *p*-values, SmartPLS uses a bias-corrected and accelerated (BCa) bootstrap method to construct confidence intervals around the parameter estimates [51]. The confidence intervals are adjusted for potential bias and skewness in the bootstrap distribution. If the confidence interval for a parameter estimate does not include zero, the estimate is considered statistically significant at the chosen significance level (e.g., 5% for *p* < 0.05) [53].

Consistent with the descriptive statistics in Table 1, the PLS model shows that female (vs. male) gender is predictive of a significantly higher level of individualizing moral foundation values (β = 0.45, *p* < 0.01), as well as a lower level of general financial literacy (β = –0.46, *p* < 0.01) and financial literacy about cryptocurrencies (β = –0.64, *p* < 0.01). However, of all the variables, only individualizing moral foundations (β = 0.19, *p* < 0.01) and binding moral foundations (β = 0.22, *p* < 0.04) further predict, statistically significantly, the outcome variable: the moral judgment of the wrongness of tax evasion. Yet, of these two variables, only individualizing moral foundations can be interpreted to explain females’ more negative judgment of tax evasion. This is because, even if binding moral foundations also predict a harsher judgment of tax evasion, females had a lower (M_females_ = 3.54), not higher, level of binding moral foundations than males (M_males_ = = 3.65) (albeit that this difference was not statistically significant). Clearly, the higher individualizing moral foundations of females dominate the effect of lower binding moral foundations, and make females’ moral judgment of tax evasion, as a net effect, more negative than males’. 

Of the other variables, income also had a marginally significant impact (β = 0.11, *p* = 0.09) on the judgment of tax evasion, suggesting that higher incomes lead to a harsher judgment of tax evasion.

As robustness checks, we estimated another PLS model with an alternative specification (Appendix A, Table A1 and Table A2) as well as a set of ordinary linear regression models (Appendix B, Table A3, Table A4 and Table A5).

**Figure A1:**
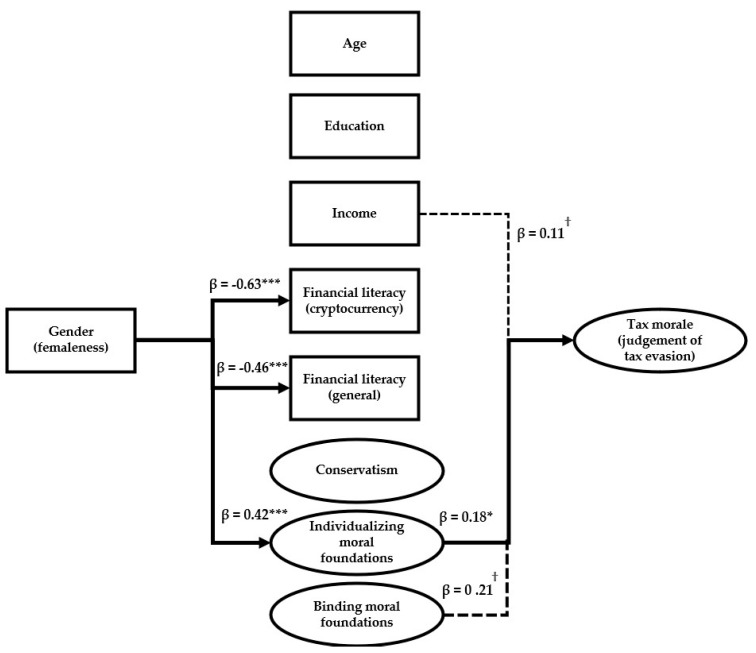
Statistically significant path coefficients from an alternative specification of the PLS model Notes. The figure shows the statistically significant PLS path coefficients. In this alternative specification of the PLS model (compared to the model in Figure 1), those question items were dropped from the latent variables “Individualizing moral foundations” and “Binding moral foundations” that had the lowest factor loadings with the variables. ^†^ *p* < 0.10, * *p* < 0.05, *** *p* < 0.001.

In the PLS model with an alternative specification, we dropped one item of each of the multi-item predictor variables, which had a Cronbach’s alpha of less than 0.9 (individualizing and binding moral foundations). The results of this alternative model (Figure A1 and Table A1 in Appendix A) are fully in line with the main model (Table 1 above). That is, female gender was positively associated with individualizing moral foundations and negatively associated with financial literacy (both general and cryptocurrency-related). Yet, of these, only individualizing moral foundations were further associated with tax morale. 

When it comes to the regression analyses, the results were also similar. Male gender predicts (Table A3 in Appendix B) a lower level of individualizing moral foundations (b = −0.206, SE = 0.101, *p* = 0.021) and a higher level of general financial literacy (b = 0.789, SE = 0.210, *p* < 0.001) and cryptocurrency-related literacy (b = 1.249, SE = 0.236, *p* < 0.001). Of these, only individualizing moral foundations further predict tax morale in terms of judged wrongness of tax evasion (Table A4: b = 0.250, SE = 0.133, *p* = 0.031). Binding moral foundations also predict tax morale (b = 0.281, SE = 0.116, *p* = 0.009), but there is no significant difference in binding moral foundations of males vs. females (b = 0.112, SE = 0.151, *p* = 0.231). In a further regression analysis with the interaction terms of gender and moral foundations included (Table A5), neither of the two interaction effects were statistically significant. This implies that the within-gender variance in moral foundations (i.e., the fact that some females, for instance, exhibit higher individualizing moral foundations and other females less high) does not have a significant ability to explain the tax morale differences of females vs. males. That is, rather than within-gender variance, it seems to be the across-gender variance in moral foundations that has most predictive ability of individuals’ tax morale.

Additionally, a posteriori power analysis, based on the observed effect sizes and sample distributions between male and female respondents, was conducted to assess the study’s statistical power. Given the sample sizes of 134 males and 109 females, this analysis aimed to determine the likelihood of correctly rejecting the null hypothesis. In two alternative power calculations, the Type I/II error rate was set at *α* = 0.01 or *α* = 0.001, corresponding to the level of significance of the difference in the mean judgment of tax evasion across female vs. male respondents (see Table 1). With *α* = 0.01 (*α* = 0.001), the post hoc power was calculated as 88.5% (68.6%).

## 5. Discussion

In this paper, we fill the gap in the existing literature on tax morale by examining the impact of gender differences on tax morale. Recent studies have also noted the importance of examining tax morale in relation to cryptocurrency [54,55]. However, most of these studies focused on country-level economic and governance factors to explain corporate tax morale of firms, regarding cryptocurrencies [56,57]. Instead, we are unaware of any previous studies on cryptocurrency-related tax morale of individual citizens. Moreover, we extend the literature on tax morale in general, by considering differences in gender. Additionally, this study enriches our knowledge of the interplay of moral values and gender as they relate to tax morale in general as well as the particular context of cryptocurrency. Previous studies, for instance, by Gagarina et al. [58], have shown that individualizing moral foundations predict a more positive attitude towards cryptocurrencies, with binding moral foundations showing the opposite. In their study, gender also predicted people’s attitudes towards seeing cryptocurrencies as legitimate currencies. However, the present research also extends the study of Gagarina et al. [58], by focusing on moral judgements of tax evasion with cryptocurrencies, rather than general attitudes towards cryptocurrencies per se.

The finding that women made harsher judgments of tax evasion aligns with past research showing that women exhibit a higher tax morale and greater disapproval of and more negative attitudes towards tax evasion (e.g., [13,14,15,16]). Furthermore, the results support recent speculation that distinct moral orientations may underpin men and women’s thinking on tax matters [13]. The present study provides empirical evidence of this notion, finding that women’s greater endorsement of individualizing moral values focused on fairness and caring predicted their more negative judgments of tax evasion. This also fits with the theory describing gender differences in moral reasoning, with women tending to emphasize empathy, relationships, and avoiding harm (e.g., [31,39]). 

By linking the gender differences in moral foundations to economic judgments, this study also expands the explanatory relevance of moral foundations theory [30] to the behavioral economics domain. Indeed, the current results demonstrate that the gender difference in individualizing moral foundation values predicted moral assessments of misconduct in an economic, or financial context. This application of the moral foundations theory responds to previous calls to test this theory’s real-world predictive validity.

These findings also have potential policy implications, especially for communication interventions aimed at improving tax compliance. Globally, approximately two thirds of companies are owned by men [59]. However, women are more likely than men to operate small, self-employed, or one-person companies. Since this study found that women and men seem to use different intuitions when judging tax evasion, compliance interventions could consider targeted messaging and engagement strategies based on these gendered patterns of business ownership. For example, tax authorities may want to emphasize the themes of societal benefits and fairness when communicating with self-employment-based or small businesses, while deterrence messaging highlighting audit risks and penalties may resonate more with the male owners of large corporations.

Furthermore, the framing of informational interventions has been shown to have strong interaction effects with gender. Hasseldine and Hite [17] showed that one and the same message about tax compliance can be interpreted differently by men and women depending on how it is framed. This indicates that gender-tailored policy messaging and interventions may be needed to improve tax morale across genders. Our findings add to their work by identifying the gender differences in specific moral value orientations and judgments, which should be considered when crafting policies to enhance tax compliance. Previous studies have shown that informational moral interventions can have gender-specific effects [60].

As one limitation of the present study, we recognize that the results may be biased by potentially differing levels of social desirability-related response patterns among females vs. males. That is, if representatives of one gender have a greater likelihood to respond to survey questions about tax morale in a socially desirable manner, then the observed differences in tax morale across the two genders may be partly explained by that social desirability (rather than gender itself, or the focal psychographic factors). Unfortunately, with the current data, we cannot examine the presence of such social desirability bias. Nevertheless, what implies that social desirability bias is not likely to be a serious concern is the fact that the two genders differed in none of the background variables available in the data (age, income, education, political orientation). Based on this, there is no reason to believe that the two genders would have differed systematically with respect to their intrinsic motivation to give socially responsible answers to survey questions either.

At any rate, the gender differences found here merit further confirmation and exploration in future research. For example, future studies could examine in more depth how specific types of financial experience shape the moral judgments of males versus females in the context of cryptocurrency tax compliance and evasion. Furthermore, while not reported in detail in this paper, further exploratory analyses, modeling the male and female sub-samples separately, pinpoint some additional ideas for future investigation. The results can be viewed from in Appendix C (Table A6 and Table A7). Specifically, among male subjects, the level of binding moral foundations had a significant positive association with how wrong an individual male judged the tax evasion to be. Also, knowledge of stock trading and cryptocurrency trading, education, and income had marginally significant associations with males’ moral judgments. These effects were not found for females, however. This can be interpreted as meaning that there is more unexplainable within-gender variance in moral judgments among females than males. That is, among males, those men who judge tax evasion more negatively are the ones who have above-average binding moral foundation values and above-average cryptocurrency literacy—but among females, such associations do not appear to exist. These different, within-gender patterns should be further examined in future research.

## Figures and Tables

**Table 1:** Descriptive statistics of studied variables across females vs. males.

	Females		Males		*t*-Test for Independent Means
	Mean	St. Dev.	Mean	St. Dev.	t Value, *p* Value
Outcome variable					
Tax morale (judgment of wrongness of tax evasion)	5.22	1.41	4.50	1.56	−3.70, <0.001 ***
Psychographic variables					
Individualizing moral foundations	4.65	0.66	4.45	0.86	−2.05, 0.04 *
Binding moral foundations	3.54	1.18	3.67	1.16	0.86, 0.39
Political orientation (conservatism)	3.44	1.84	3.77	1.70	1.30, 0.19
Financial literacy (general)	4.79	2.08	5.77	2.10	3.64, <0.001 ***
Financial literacy (cryptocurrency)	4.05	2.53	5.63	2.20	5.19, <0.001 ***
Demographic variables (other than gender)					
Education	4.12	1.30	3.90	1.33	1.28, 0.20
Income	6.05	3.05	5.81	3.09	0.61, 0.55
Age	40.9	11.9	38.5	13.3	1.46, 0.15

Notes: In Table 1, we report the mean, standard deviation, and the results of the *t*-test of the main variables used in this study. * *p* < 0.05, *** *p* < 0.001.

**Table 2:** Correlation table of measured variables.

	1.	2.	3.	4.	5.	6.	7.	8.	9.	10.
1. Gender	N/A									
2. Judgment of wrongness of tax evasion	−0.232 **	(0.90)								
3. Individualizing moral foundations	−0.131 *	0.164 *	(0.62)							
4. Binding moral foundations	0.055	0.198 **	0.110	(0.89)						
5. Financial literacy (general)	0.229 **	0.020	−0.058	0.134 *	N/A					
6. Financial literacy (cryptocurrency)	0.318 **	−0.032	−0.056	0.110	0.745 **	N/A				
7. Political orientation (conservatism)	0.084	0.057	−0.195 **	0.643 **	0.123	0.097	(0.94)			
8. Age	0.093	0.071	−0.002	0.035	−0.003	−0.222 **	0.027	N/A		
9. Education	−0.082	0.83	−0.074	0.153 *	0.300 **	0.193 **	0.038	0.076	N/A	
10. Income	0.039	0.117	0.043	−0.005	0.168 **	0.101	−0.028	0.045	0.176	N/A

Notes: The diagonal indicates the Cronbach’s alphas for multi-item variables. * *p* < 0.05, ** *p* < 0.01.

## Data Availability

The data presented in this study are available on request from the corresponding author, Jori Grym.

## Appendix A. Robustness Check with a PLS Model with Alternative Specification of Multi-Item Latent Variables (Moral Foundations)

**Table A1:** The effects of gender (male) on all other variables—from an alternative specification of the PLS model.

Predicting Variable	Predicted Variable	β	SD	t	*p* Value
Gender (maleness)	Age	−0.187	0.127	1.472	0.141
	Education	−0.165	0.126	1.307	0.252
	Income	−0.078	0.128	0.612	0.541
	Financial literacy (cryptocurrency)	0.638	0.120	5.306	0.000 ***
	Financial literacy (general)	0.459	0.122	3.723	0.000 ***
	Political orientation (conservatism)	0.168	0.129	1.299	0.194
	Individualizing moral foundations	−0.421	0.112	3.757	0.000 ***
	Binding moral foundations	0.040	0.247	0.160	0.873

Notes: In this alternative specification of the PLS model (compared to the model in Figure 1), those question items were dropped from the latent variables “Individualizing moral foundations” and “Binding moral foundations” that had the lowest factor loadings with the variables. *** *p* < 0.001.

**Table A2:** The effects of all predictor variables on tax morale (judgment of wrongness of tax evasion)—from an alternative specification of the PLS model.

Predicted Variable	Predicting Variable	β	SD	t	*p* Value
Tax morale (judgment of wrongness of tax evasion)	Age	0.048	0.069	0.694	0.487
	Education	0.079	0.070	1.128	0.259
	Income	0.110	0.063	1.739	0.082 ^†^
	Financial literacy (cryptocurrency)	0.019	0.098	0.195	0.846
	Financial literacy (general)	−0.084	0.112	0.747	0.455
	Political orientation (conservatism)	−0.033	0.082	0.400	0.689
	Individualizing moral foundations	0.183	0.073	2.498	0.013 *
	Binding moral foundations	0.211	0.110	1.925	0.054 ^†^

Notes: In this alternative specification of the PLS model (compared to the model in Figure 1), those question items were dropped from the latent variables “Individualizing moral foundations” and “Binding moral foundations” that had the lowest factor loadings with the variables. ^†^ *p* < 0.10, * *p* < 0.05.

## Appendix B. Robustness Check with a Series of Linear Regression Analyses

**Table A3:** The effects of gender (male) on all other variables—from linear regression analyses.

Mediating Variable as Outcome Variable	Predictor Variable—Gender	b	SE	t	*p* Value	Partial Eta Squared
Age	Intercept	40.862	1.218	33.537	<0.001	0.825
	Gender (male)	−2.325	1.646	−1.412	0.079	0.008
Education	Intercept	4.119	0.126	32.709	<0.001	0.817
	Gender (male)	−0.218	0.170	−1.280	0.101	0.007
Income	Intercept	6.046	0.295	20.522	<0.001	0.638
	Gender (male)	−0.235	0.398	−0.591	0.278	0.001
Financial literacy (cryptocurrencies)	Intercept	3.147	0.175	17.996	<0.001	0.575
	Gender (male)	1.249	0.236	5.286	<0.001	0.105
Financial literacy (general)	Intercept	3.725	0.155	23.974	<0.001	0.706
	Gender (male)	0.789	0.210	3.757	<0.001	0.056
Political orientation (conservatism)	Intercept	3.440	0.174	19.807	<0.001	0.621
	Gender (male)	0.272	0.235	1.158	0.124	0.006
Individualizing moral foundations	Intercept	4.654	0.075	62.246	<0.001	0.942
	Gender (male)	−0.206	0.101	−2.041	0.021	0.017
Binding moral foundations	Intercept	3.538	0.112	31.654	<0.001	0.807
	Gender (male)	0.112	0.151	0.739	0.231	0.002

**Table A4:** The effects of all predictor variables on tax morale (judgment of wrongness of tax evasion)—from a linear regression analysis.

Outcome Variable—Tax Morale	Mediating Variable as Predictor Variable	b	SE	t	*p* Value	Partial Eta Squared
Tax morale (judgment of wrongness of tax evasion)	Intercept	2.129	0.810	2.628	0.005	0.029
	Age	0.007	0.008	0.926	0.177	0.004
	Education	0.086	0.079	1.095	0.138	0.005
	Income	0.058	0.032	1.815	0.036	0.014
	Financial literacy (cryptocurrency)	0.001	0.079	0.014	0.495	0.000
	Financial literacy (general)	−0.072	0.093	−0.773	0.220	0.003
	Political orientation (conservatism)	−0.037	0.075	−0.495	0.311	0.001
	Individualizing moral foundations	0.250	0.133	1.877	0.031	0.015
	Binding moral foundations	0.281	0.116	2.415	0.009	0.025

**Table A5:** The effects of all predictor variables on tax morale, with interaction terms of moral foundations and gender—from a linear regression analysis.

Dependent Variable	Independent Variable	b	SE	t	*p* Value	Partial Eta Squared
Tax morale (judgment of wrongness of tax evasion)	Intercept	2.755	1.185	2.325	0.011	0.023
	Age	0.007	0.008	0.890	0.188	0.003
	Education	0.043	0.078	0.548	0.292	0.001
	Income	0.052	0.032	1.624	0.053	0.011
	Financial literacy (cryptocurrency)	0.054	0.080	0.669	0.252	0.002
	Financial literacy (general)	−0.059	0.092	−0.643	0.261	0.002
	Political orientation (conservatism)	−0.042	0.074	−0.562	0.287	0.001
	Individualizing moral foundations	0.180	0.222	0.813	0.208	0.003
	Binding moral foundations	0.295	0.150	1.965	0.025	0.017
	Gender (male)	−0.783	1.317	−0.594	0.277	0.002
	Gender X Individualizing moral foundations	0.020	0.266	0.074	0.471	0.000
	Gender X Binding moral foundations	0.005	0.168	0.031	0.488	0.000

## Appendix C

**Table A6:** The effects of all predictor variables on tax morale (judgment of wrongness of tax eva-sion)—from a PLS model with female respondents only.

Dependent Variable	Independent Variable	β	SE	t	*p* Value
Tax morale (judgment of wrongness of tax evasion α = 0.886)	Age	0.076	0.085	0.899	0.184
	Education	−0.067	0.102	0.656	0.256
	Income	0.064	0.085	0.752	0.226
	Financial literacy (cryptocurrency)	−0.008	0.118	0.070	0.472
	Financial literacy (general)	0.049	0.141	0.346	0.365
	Political orientation (conservatism)	0.014	0.120	0.120	0.452
	Individualizing moral foundations	0.173	0.153	1.126	0.130
	Binding moral foundations	0.232	0.143	1.625	0.052

**Table A7:** The effects of all predictor variables on tax morale (judgment of wrongness of tax eva-sion)—from a PLS model with male respondents only.

Dependent Variable	Independent Variable	β	SD	t	*p* Value
Tax morale (judgment of wrongness of tax evasion α = 0.886)	Age	0.063	0.094	0.685	0.247
	Education	0.122	0.094	1.311	0.095
	Income	0.134	0.090	1.500	0.067
	Financial literacy (cryptocurrency)	0.155	0.109	1.425	0.077
	Financial literacy (general)	−0.200	0.125	1.603	0.055
	Political orientation (conservatism)	−0.086	0.101	0.855	0.196
	Individualizing moral foundations	0.122	0.142	0.856	0.196
	Binding moral foundations	0.242	0.113	2.143	0.016
